# Probiotic *Bacillus Clausii* Protects against Amoxicillin-evoked Reproductive Dysfunction through Oxidative Stress Modulation

**DOI:** 10.1007/s12602-025-10891-x

**Published:** 2026-02-05

**Authors:** Nadia A. El-Fahla, Nahla S. El-Shenawy, Noran M. Tawfik

**Affiliations:** Zoology Department, Faculty of Science, Suez Canal University, Ismailia, Egypt

**Keywords:** Antibiotics, Antioxidants, Oxidative stress, Histopathology, mTOR, Gene expression

## Abstract

Antibiotics are frequently used in livestock, aquaculture, and poultry, resulting in their accumulation in these animals and the surrounding environment. This contamination can present health risks to humans. Research on the harmful effects of antibiotics, especially their potential to disrupt hormones in the male reproductive system, is still in its early phases. This study investigated the impact of exposure to the antibiotic amoxicillin (AMO) on male reproductive health. The aim is to shed light on the potential dangers antibiotics pose to male reproductive health and to emphasize how *Bacillus clausii* may help protect against the toxic effects of AMO. The study included four groups treated via gavage: control animals, a supplemented group receiving *B. clausii* (1.25 ml, 1 × 10^9 CFU per mouse per day), a group treated with AMO at 30 mg/kg daily, and a fourth group that received AMO and *B. clausii*. After 45 days of treatment, blood samples were collected to assess serum activities of superoxide dismutase and catalase and levels of reduced glutathione, malondialdehyde, and testosterone. Testicular tissue samples were analyzed for mechanistic target of rapamycin **(**mTOR) gene expression and histological changes. This study demonstrates that exposure to AMO negatively impacts male reproductive health by increasing oxidative stress, reducing testosterone levels, causing testicular damage, and downregulating the mTOR expression level. However, supplementation with *B. clausii* mitigates these harmful effects, likely due to its antioxidant properties. The probiotic reduced oxidative stress markers, improved testosterone levels, and alleviated testicular lesions, thereby upregulating the mTOR expression level, which suggests its potential protective role against antibiotic-induced toxicity.

## Highlights

- Histopathological damage and oxidative stress markers were affected by AMO.
- We explored amoxicillin-induced reproductive toxicity.
- We demonstrated, for the first time, that Bacillus clausii can protect the male reproductive system from this antibiotic toxicity.
- We linked the protection to antioxidant activity and upregulation of mTOR gene expression — a novel mechanistic insight.
- AMO disrupted cellular metabolism and impaired spermatogenic regulation.
- Probiotics can modulate the PI3K/AKT/mTOR pathway, reduce autophagy, and support Leydig cell function.

## Introduction

Beta-lactam antibiotics, such as penicillins and cephalosporins, are commonly used to treat bacterial infections like urinary tract infections and pneumonia. They work by disrupting bacterial cell wall synthesis, resulting in cell death [47]. Beta-lactam antibiotics like amoxicillin (AMO) offer therapeutic benefits, but concerns about their side effects exist. Amoxicillin can cross the placenta in early pregnancy, and animal studies link its use to fatal growth issues and skeletal malformations. Caution is vital when using these antibiotics during pregnancy to reduce developmental risks.

Amoxicillin is considered safe for treating bacterial infections in both humans and animals [14]. However, it is now recognized as an “emerging contaminant” in the environment [2]. This leads to widespread exposure for humans and animals [14] and its excessive use in livestock has resulted in drug residues contaminating food products like milk, eggs, and meat [6]. AMO residues are present in hospitals, factories, and pharmaceutical effluents, as well as in various aquatic ecosystems, including groundwater and wastewater [25, 41]. More than 80% of AMO is eliminated via urine within 2 h, and its environmental presence can cause significant ecological issues [15]. In hospital wastewater, AMO concentrations can reach 1,172,000 ng/L, while urban wastewater varies from 66 to 5,230 ng/L, with a European maximum limit of 78 ng/L [16].

Amoxicillin is widely used in food-producing animals to treat bacterial infections and is available as oral suspensions, boluses, or injectables based on species and weight. Monitoring animal products for antibiotic residues is crucial to prevent antimicrobial resistance and ensure food safety [40]. AMO is rapidly absorbed orally in mice, rats, and dogs, reaching peak plasma levels within 1–2 h. Its plasma levels do not increase proportionally with dosage, indicating a saturable absorption mechanism. The drug distributes well into tissues, with low plasma protein binding, and is quickly eliminated, exhibiting short half-lives and no accumulation. These characteristics underscore the importance of tailored dosing strategies for effective therapy [61].

Amoxicillin is mainly excreted unchanged in urine, with metabolism producing degradation products like amoxicillin acid (AMA) and diketopiperazine-2′,5′-dione (DIKETO) [62]. While some metabolites have been identified in vitro, their in vivo formation is uncertain. Studies in humans and pigs confirm the presence of AMA and its epimer, as well as amoxicillin-(2R)-piperazine-2′,5′-dione [23, 44]. Pharmacokinetic comparisons show that AMA and DIKETO appear in plasma shortly after intravenous administration, while they are detected after oral dosing only following amoxicillin absorption [45]. AMA’s longer elimination half-life than AMO suggests it is cleared more slowly, likely through glomerular filtration rather than active secretion. These findings enhance our understanding of AMO’s metabolism and excretion, which is crucial for improving its use in clinical and veterinary settings.

An in vitro study indicated that AMO can cause DNA damage, likely through oxidative stress, and the damage appears reversible. This suggests that the observed damage may result indirectly from oxidative free radicals [4]. Other genotoxicity tests, including chromosomal aberration and micronucleus assays, found no evidence of genetic toxicity [27]. These findings suggest that while AMO may induce temporary DNA damage at high concentrations, it does not appear to have lasting mutagenic effects. The creation of reactive oxygen species (ROS) correlated with DNA damage, reinforcing the hypothesis that oxidative stress is involved [32].

Fahmy et al. [18] indicated that AMO-clavulanic acid negatively affects the male reproductive system in mice. The treatment led to increased sperm abnormalities and significant histopathological changes in testicular tissue, suggesting potential toxicity to male reproductive health. Moreover, Sayem et al. [49] provide a detailed pharmacodynamic profile of AMO against multidrug-resistant *Staphylococcus aureus* and *Staphylococcus pseudintermedius* in canine clinical isolates. The findings highlight AMO’s efficacy in targeting these gram-positive pathogens, although resistance remains a concern. The study underscores the importance of optimizing dosage strategies to enhance AMO’s effectiveness while minimizing the risk of resistance development.

Amoxicillin was selected as the β-lactam antibiotic in this experiment due to its widespread clinical use, oral availability, and previously reported ability to induce oxidative and reproductive disturbances, making it a relevant model for evaluating the protective efficacy of *Bacillus clausii* (BAC). It is an aerobic, rod-shaped, Gram-positive, alkaline-tolerant, non-pathogenic bacterium that produces endospores, and its strains proliferate in different environmental conditions, including the human gastrointestinal tract [59]. The strains produce durable spores that can endure harsh environmental conditions, including alkalinity, droughts, ultraviolet radiation, elevated temperatures, starvation, and exposure to harsh physical or chemical agents. Upon re-establishing a tailored environmental situation, the spores germinate and transform into vigorous vegetative cells, prepared to flourish and reproduce [13, 48].

BAC spores are metabolically inactive and exhibit better resistance to the acidity of bile salts or stomach HCl [17, 24] They also seem to exhibit a greater tolerance to heat treatments (during pharmaceutical processing or the production of functional meals), especially when fortified with a protectant like trehalose sugar [7]. BAC can repair damaged intestinal membranes and restore vitamin deficiencies in the gut induced by antibiotics or chemotherapeutic drugs. Nonetheless, the most notable advantage of the bacterium is its probiotic trait of antibiotic resistance, a distinctive property that cannot be genetically acquired, adopted, or transferred to other pathogens inhabiting the same environment [48].

Although Dai et al. [14] reported the protective role of *Bacillus coagulans* in reproductive impairment associated with microbiota disturbance, the influence of probiotic *Bacillus clausii* on antibiotic-induced reproductive toxicity has not yet been elucidated. Therefore, this study aims to investigate the reproductive toxicity caused by AMO exposure and explore the protective effects of *Bacillus clausii*. By analyzing oxidative stress markers, testosterone levels, mechanistic target of rapamycin (mTOR) gene expression, and testicular tissue structure, the research provides a deeper understanding of how antibiotics may impair male fertility and whether probiotic intervention can help counteract these effects. The findings also highlight the broader implications of environmental antibiotic contamination on reproductive health.

## Materials and Methods

### Animals and Experimental Protocol

The experiments were designed with 24 male Wistar albino mice (1 month old, 25–27 g). The mice were obtained from the Laboratory Animal House at the Faculty of Veterinary Medicine, Suez Canal University, Ismailia, Egypt. They were housed three males per cage, at a room temperature of 24 ± 2 °C and exposed to natural daylight cycles. Water and food were provided *ad libitum*. A suitable environment was maintained, free from noise and cold, which helped reduce stress and ensured proper hygiene for the mice during the experiment. Before the experiment, the mice underwent a one-week acclimatization period. All experimental protocols were conducted following the ethical guidelines for the use of laboratory animals established by the Faculty of Veterinary Medicine, Suez Canal University, Egypt (SCU-VET-AREC-R-2025013).

The mice were permitted free access to a pelleted diet and tap water. The mice were divided into four groups (*n* = 6/group) and treated daily by gavage for 45 days. The 1st group considered the control group received saline as vehicles, and the 2nd group administered BAC (1.25 ml, 1 × 10^9^ CFU per mouse/day) as a commercial probiotic (Enterogermina^®^, Sanofi, Turkey) [43]. The 3rd group received AMO as a commercial antibiotic (E-MOX^®^, E.I.P.I.CO., Egypt) at 30 mg/kg [3]. The 4th group was simultaneously administered AMO 30 mg/kg and BAC 1.25 ml, 1 × 10^9^ CFU per mouse.

The selected amoxicillin dose of 30 mg/kg was based on previous experimental studies that demonstrated this dose induces measurable oxidative stress and reproductive impairment without causing systemic toxicity or high mortality [3]. Therefore, 30 mg/kg is considered appropriate for producing reproducibility-toxic effects that are detectable and biologically relevant. Regarding *Bacillus clausii*, the administered dose was based on its clinically used probiotic formulation and supported by previous studies demonstrating beneficial effects at 1 × 10⁹ CFU/day in restoring gut microbial balance and alleviating oxidative and inflammatory conditions [43]. This dose is within the therapeutic range commonly recommended for human probiotic supplementation, and has been shown to be safe and effective in modulating systemic oxidative markers in experimental models.

### Body and Testis Weights

Body weights were recorded at the start and conclusion of the experiment for the mice. The gonadosomatic index (GSI%) of the testes was determined using the formula: (testis weight at the end of the experiment / final body weight) × 100 [54].

### Sampling Collection

After 45 days, the male mice were anesthetized by inhalation of tetrahydrofuran. Two blood samples for each mouse were obtained from the retro-orbital venous plexus of the eye; the first one was collected and left for clot formation to separate the serum. The separated sera were stored at − 80 °C and used for the estimation of antioxidant parameters and lipid peroxidation. The second one was collected in EDTA tubes. They were centrifuged at 4 ^◦^C and 3000 g for 10 min to obtain plasma that was kept at -80 ^◦^C for testosterone analysis. Testicular tissue samples (six per group) from each mouse were fixed in 10% neutral buffered formalin for histopathological analysis. Another testis sample was frozen in RNA later and then tested for mRNA expression of mTOR.

### Antioxidant and MDA Assays

Superoxide dismutase (SOD) activity was measured using an ELISA kit (Catalog No. SL0513Mo; SunLong Biotech Co., USA). Catalase (CAT) activity was assessed with another ELISA kit (Catalog No SL0747Mo). Glutathione (GSH) levels were determined using a mouse glutathione ELISA kit (Catalog No SL0919). Malondialdehyde (MDA) levels were measured with an ELISA kit (Catalog No SL0370Mo). Assay procedures followed the manufacturer’s protocol.

### Testosterone Assay

Mouse plasma from six samples per group was analysed for testosterone levels using a specific ELISA kit (Catalog No SL0657Mo). The assay procedures were carried out according to the manufacturer’s protocol.

### Quantitative Real-time PCR

Testicular tissue specimens from all groups of mice were obtained for gene expression analysis of the mTOR gene. Total RNA extraction was performed using the ABT Total RNA kit (Catalog No ABT002, Applied Biotechnology, Egypt) following the manufacturer’s guidelines. Following the manufacturer’s instructions, the isolated mRNA was subjected to reverse transcription to cDNA with the ABT 2x RT Mix Oligo kit (Catalog No AMP11, Applied Biotechnology, Egypt). Gene expression analysis was assessed using the Applied Biosystems StepOne™ Real-Time PCR System. The housekeeping gene used for data normalization was GAPDH. The expression of mTOR and the housekeeping genes in the testicular tissues was detected with the primers listed in Table 1. Thermal cycling and fluorescence detection were performed using the ABT 2x qPCR Mix (SYBR) high ROX mix (Catalog No AMP04, Applied Biotechnology, Egypt). The thermal cycling conditions consist of 40 cycles, including 10 min at 95 °C, followed by 15 s at 95 °C, 30 s at 55 °C, and finalized by 30 s at 72 °C. The melting curves were investigated to verify the specificity of the amplification reaction. All reactions were carried out in triplicate for the target primer sequences. The obtained results were assessed using the 2^−ΔΔCt^ method [34].

**Table 1:** Primer sequences for qRT-PCR

Gene	Forward (5′–3′)	Reverse (5′–3′)
mTOR	GGAGTCTTGTGGCATTTGGA	TGAGTGGAGTAGGGGAGCAG
GAPDH	TGGCCTTCCGTGTTCCTAC	GAGTTGCTGTTGAAGTCGCA

### Histopathological Assessment of the Testis

Testicular tissues from the various experimental groups were preserved in 10% (v/v) formalin prepared in phosphate-buffered saline (PBS). After washing with tap water, they were treated with 70% and 100% ethyl alcohol. The samples were rinsed in xylene for clarity, embedded in paraffin at 60 °C for 24 h, and sectioned at 5 μm using a rotary microtome (Leica Biosystems). The sections were collected on glass slides, deparaffinised, and stained with haematoxylin and eosin [5]. Then, the slides were examined under an optical light microscope (Olympus, Model: CX41RF, Olympus Corporation, Tokyo, Japan).

Histopathological changes in the testis were assessed using a semiquantitative lesion scoring system. A total of 6 slides/treatment, each from a different mouse, were examined at 200x magnification using a light microscope. The histopathological lesions were scored according to the following criteria: mild (+) for less than 25% of the area affected, moderate (++) for 25–50% of the area affected, and severe (+++) for more than 50% of the area affected [1].

### Statistical Analysis

Data from individual replicates of the control and experimental groups were pooled and analyzed using GraphPad Prism software version 8. Normality was assessed with the Shapiro-Wilk test, and homogeneity of variances was tested with Bartlett’s test. A one-way ANOVA was conducted to identify group differences, followed by Tukey’s test for post hoc comparisons. Results are presented as mean ± SE, with different superscript letters (a, b, c) indicating significant differences at *p* < 0.05. Groups with different letters are significantly different, while means with the same letter are not.

## Results

### Body Weight and Testicular GSI

Treatment with AMO and supplementation with BAC exhibited non-statistical variation in final weight compared to the control group (*p* = 0.05). Moreover, the different groups had no significant differences in GSI values. However, the BAC-supplemented group demonstrated a significant increase in GSI values compared to the AMO-treated mice (*p* < 0.03) (Table 2).

**Table 2:** Effects of *Bacillus Clausii* on body weight and gonad-somatic index in male mice exposed to amoxicillin

Group	Control	BAC (1 × 10^9^ CFU/d)	30 mg AMO /kg	30 mg AMO /kg + BAC (1 × 10^9^ CFU/d)	*P*-value
Parameters					
Initial WT (g)	25 ± 0.42	27 ± 0.50	26 ± 0.31	26 ± 0.37	0.27
Final WT (g)	28 ± 0.60	30 ± 0.60	28 ± 0.58	30 ± 0.67	0.05
GSI %	0.85 ± 0.01 ^ab^	0.86 ± 0.01^a^	0.77 ± 0.03^b^	0.85 ± 0.02^ab^	0.02

Data are presented as mean ± SE (*n* = 6 *per group*). Values in the same row with different superscripts (a and b) indicate that their corresponding means are significantly different (*p* < 0.05) based on a one-way ANOVA followed by Tukey’s multiple comparison test. Initial WT: mouse body weight in grams at zero-day, Final WT: mouse body weight in grams after 45 days, *GSI* gonad (testis)-somatic index

### Antioxidant Activity and MDA Levels

**Fig. 1:**
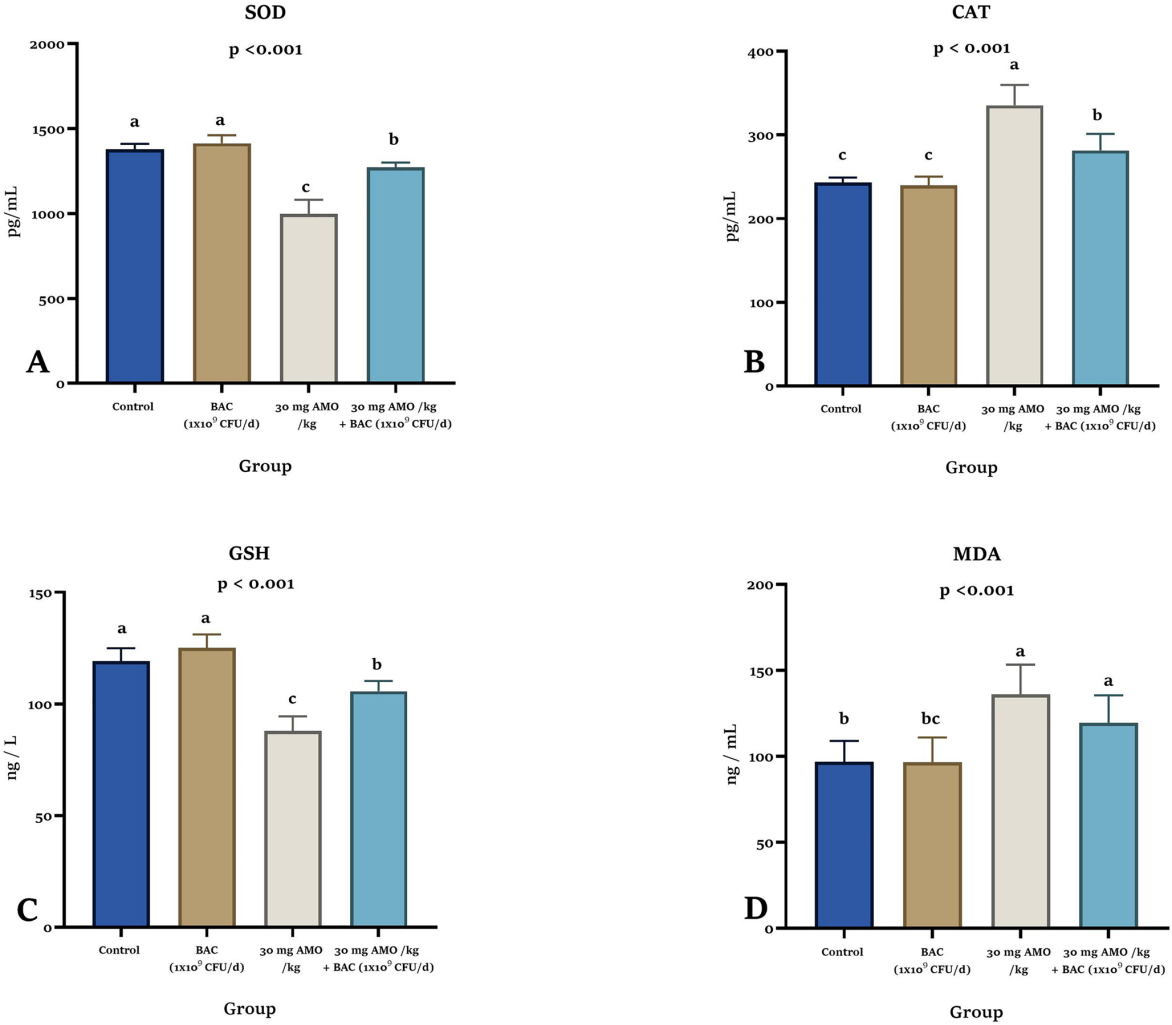
(**A-D**): A probiotic bacterium, *Bacillus clausii* (BAC), ameliorated antioxidant parameters and MDA influenced by amoxicillin (AMO) antibiotic in sera of male albino mice. (**A**) superoxide dismutase (SOD), (**B**) catalase (CAT), (**C**) glutathione reduced (GSH), and (**D**) malondialdehyde (MDA) levels. Data are presented as the mean ± SE with *n* = 6 per group, analyzed using a one-way ANOVA. Groups marked with different letters (a, b, and c) above the bars are significantly different at a p-value less than 0.05, according to post hoc Tukey’s test

Exposure to AMO significantly increased MDA levels (136.0 ± 7.0, *p* < 0.001) compared to the control (97.0 ± 4.9). AMO significantly decreased SOD (999.0 ± 34.0) and GSH (88.0 ± 2.7) and increased CAT (335.0 ± 9.9), compared to control values (1377 ± 14.0 for SOD, 119.0 ± 2.4 for GSH and 243.0 ± 2.5 for CAT), with a p-value of less than 0.001 (Fig. 1A-C). Administration of BAC to the AMO-exposed group decreased the MDA level non-significantly (120.0 ± 6.5, *p* = 0.25) while significantly (*p* < 0.001) increasing SOD (1272.0 ± 12.0) and GSH (106 ± 1.9) levels, and decreasing the CAT (281.0 ± 8.2) level compared to the AMO-treated group. Figure 1D revealed no significant differences in the levels of MDA between BAC-supplemented (97.0 ± 5.9) and control (97.0 ± 4.9) mice. Similarly, the BAC group exhibited a non-statistically significant elevation in SOD (1414.0 ± 19.0), CAT (240.0 ± 4.3) levels, and GSH (125.0 ± 2.5) content relative to the control.

### Testosterone Levels

**Fig. 2:**
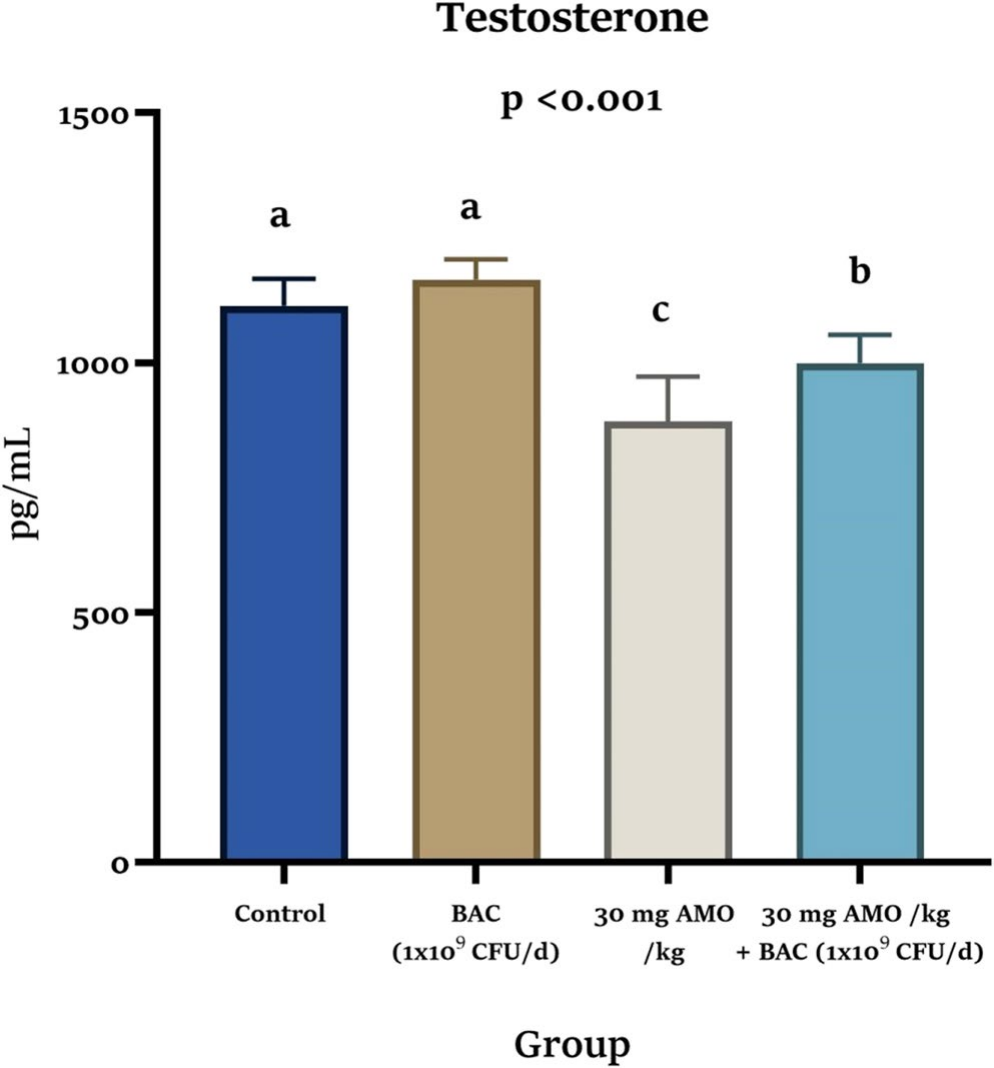
A probiotic bacterium, *Bacillus clausii* (BAC), improved testosterone levels affected by amoxicillin (AMO) antibiotic in sera of male albino mice. Data are presented as the mean ± SE with *n* = 6 per group, analyzed using a one-way ANOVA. Groups marked with different letters (a, b, and c) above the bars are significantly different at a p-value less than 0.05, according to post hoc Tukey’s test

The plasma levels of the testosterone hormone in the control group (1114.0 ± 22.0) and the BAC-protective group (1166.0 ± 17.0) exhibited a significant increase (*p* < 0.001) compared to the other groups. In contrast, the AMO-treated group had a significantly lower testosterone level (882.0 ± 36.0, *p* < 0.001) compared to the control mice. However, the administration of BAC to the AMO-treated group resulted in a significant elevation in testosterone levels (999.0 ± 23.0, *p* < 0.05) compared to the AMO-treated group (Fig. 2).

### Real-time PCR

**Fig. 3:**
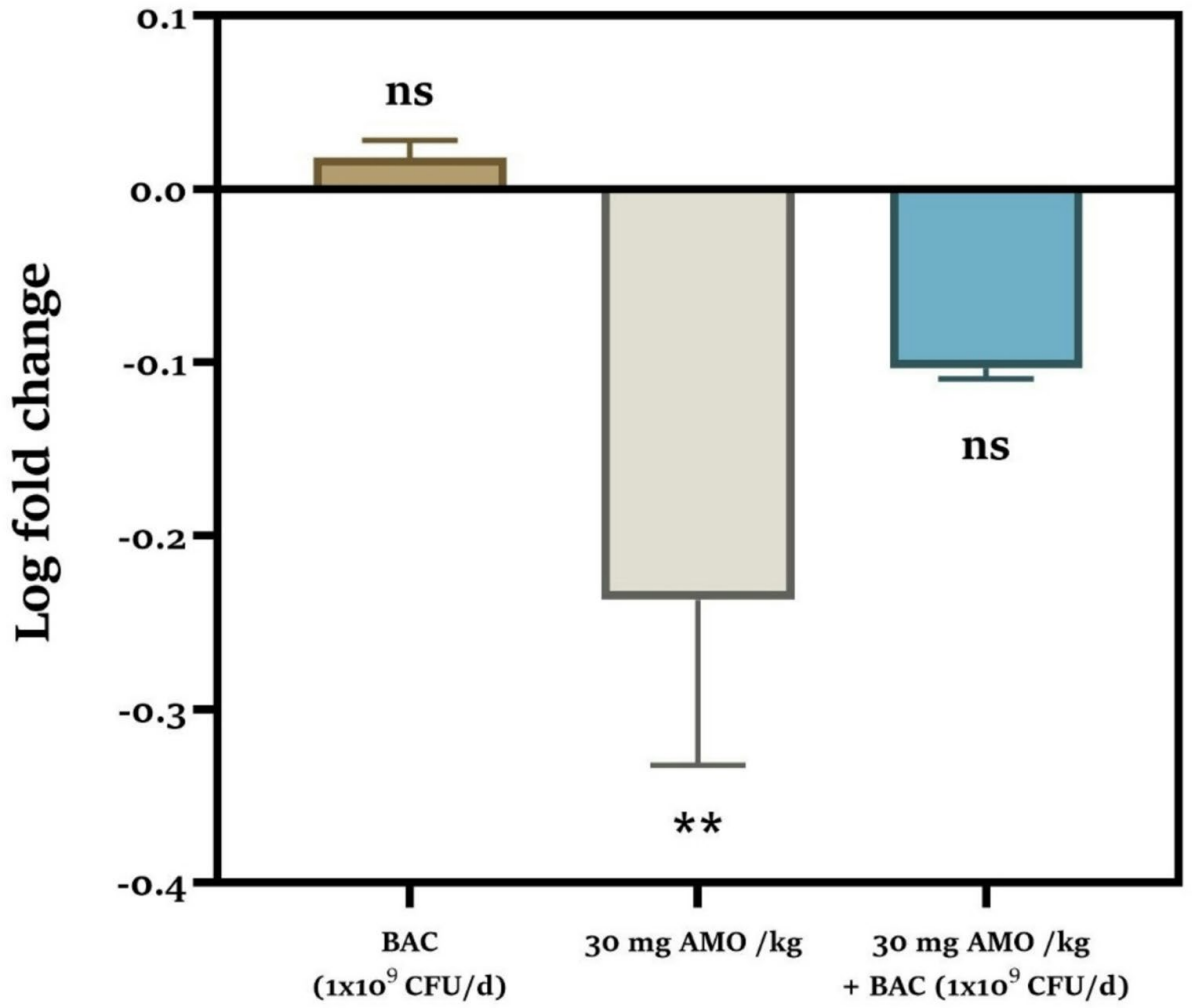
Gene expression analysis of mTOR in the testicular tissue of male albino mice represented by log fold change compared to the control group. It showed significant downregulation of the mRNA transcript level in the group exposed to AMO compared to the control. On the contrary, administration of BAC to the AMO-exposed group showed a significant upregulation in the mRNA level compared to the AMO group, indicating the ameliorative effect of the BAC. *, **, and *** denoted significant differences between groups at *P* < 0.05, *P* < 0.01, and *P* < 0.001, respectively. ns: Non-significant

The transcript level of the mTOR gene varied among different groups. The gene expression analysis is represented by a log-fold change of the transcript, as indicated in Fig. 3. The BAC group exhibited a non-statistically significant upregulation in the mRNA transcript level relative to the control. However, AMO-treated mice showed a significant downregulation of the mTOR transcript level compared to the BAC-administered group, with a p-value of less than 0.001. The AMO-exposed group demonstrated a statistically significant (*P* < 0.01) downregulation in the expression level of mTOR compared to the control animals. The administration of BAC to the AMO-exposed group showed no statistically significant variation compared to that of the control group. Administration of BAC to the AMO-exposed group statistically (*P* < 0.05) upregulated the mTOR transcript level compared to the AMO-exposed group, indicating the ameliorative effect of the BAC.

### Pathological Changes in Testes

**Fig. 4:**
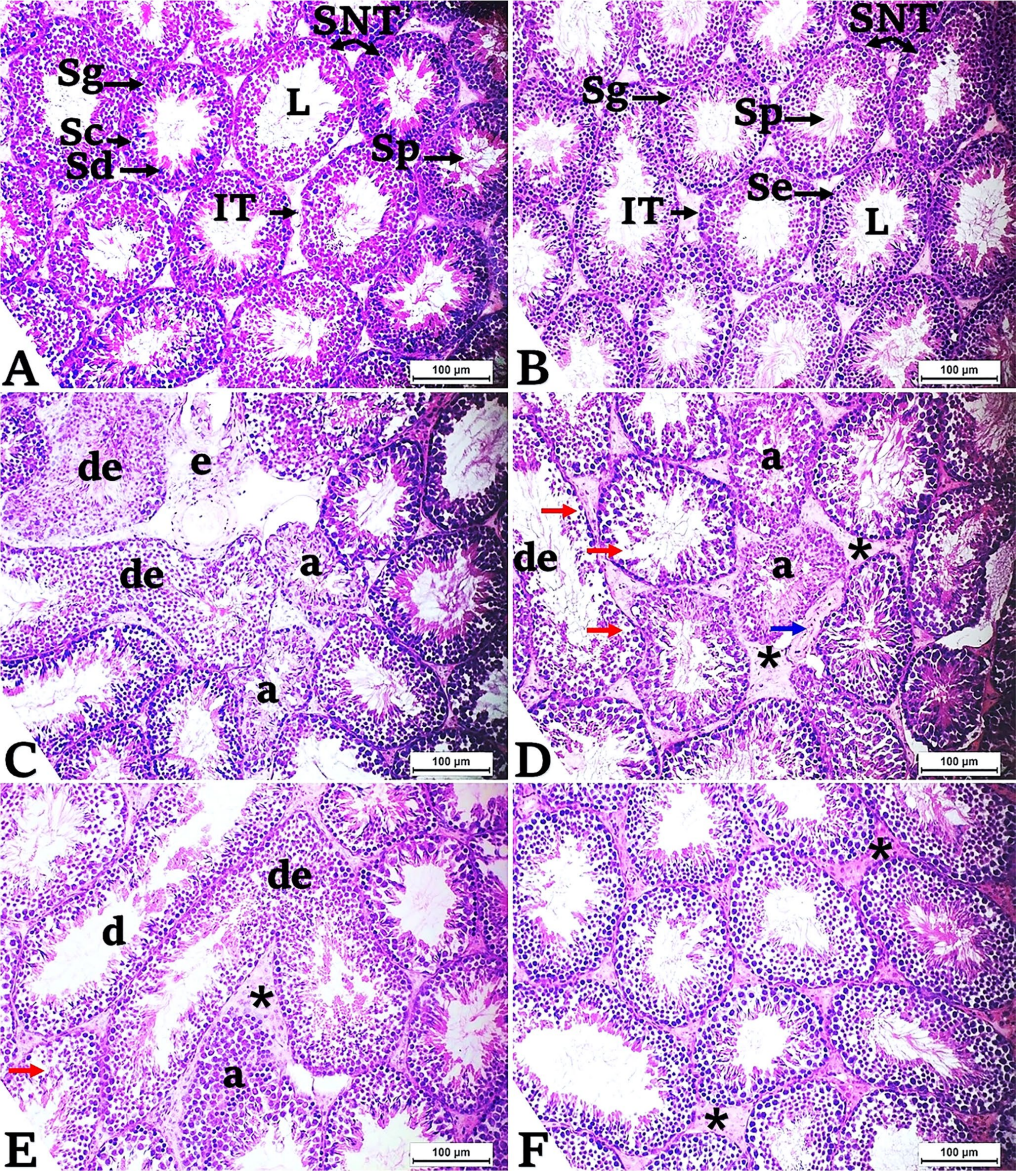
(**A-F**): Testicular cross-sections of mice, stained with H&E (x 200: bar, 100 μm). **A** and **B**, testis sections of control and BAC groups, respectively, revealed the normal histological structure of seminiferous tubules (SNT) with typical seminiferous epithelium consisting of Sertoli cells (Se) in the basement of tubules, spermatogonia (Sg), spermatocytes (Sc), spermatids (Sd), and mature sperms (Sp) can be observed clearly in the lumen (L). The seminiferous tubules are separated by numerous Leydig interstitial cells (IT). **C**, **D**, and **E**, testis sections of AMO-treated mice showed atrophied (a) deformed seminiferous tubules with an interrupted basement membrane and a narrow lumen. In addition to degenerating (de) seminiferous tubules with signs including dilation (d), a disordered arrangement, and a depletion of spermatogenic cells (red arrows). Diffusely hyperplastic interstitial cells (*) with necrotic Leydig cells, hyalinization (blue arrow), and edema (e) within the interstitial tissues. **F**, testicular section of the AMO + BAC group demonstrated improvement in mitigating AMO-induced histotoxic effects, though hyperplastic interstitial cells (*) without Leydig apoptosis persisted

The histological analysis of testicular tissues exhibited a normal histological architecture in the control and BAC-supplemented groups (Fig. 4A and B). The seminiferous tubules appeared well-organized, separated by numerous interstitial cells of Leydig, and displayed the typical arrangement of spermatogenic cells and Sertoli cells. Besides, well-defined mature spermatozoa were directed toward the lumen.

The testis sections (Fig. 4C-E) from AMO-treated mice after 45 days revealed a combination of atrophic and degenerating seminiferous tubules, along with signs of an interrupted basement membrane “degeneration/atrophy, tubular”. The degenerated seminiferous tubules exhibited histotoxic features, including dilation, hyperplasia of germ cells, and a disordered arrangement of spermatogenic cells, as well as reduced spermatogenesis and spermatozoa. In contrast, the atrophied tubules exhibited a depletion of spermatogenic cells and a slightly dilated lumen. The marked detectable lesions included diffusely hyperplastic interstitial cells, hyalinization, and edema within the interstitial tissues. The testicular profile of the AMO + BAC group suggested a potential improvement in countering the testicular histotoxic effects caused by AMO administration. However, diffusely hyperplastic interstitial cells were still present (Fig. 4F). Table 3 presents the extent of histopathological changes identified in the analyzed testicular tissues.

**Table 3:** Semi-quantitative scoring of the detectable histopathological lesions in the testis of male mice exposed to amoxicillin

Lesion	Control	BAC (1 × 109 CFU/d)	30 mg AMO /kg	30 mg AMO /kg + BAC (1 × 109 CFU/d)
Seminiferous tubules				
- Tubular Atrophy	**-**	-	+++	-
- Depletion of spermatogenic cells	-	-	+++	+
- Narrow lumen	-	-	+++	-
- Tubular Degeneration	-	-	+++	+
- Dilation	-	-	+++	+
- Hyperplasia of germ cells	-	-	+++	-
- Disordered arrangement of spermatogenic cells.	-	-	+++	-
- Reduced spermatogenesis, and spermatozoa	-	-	+++	+
Interstitial cells of Leydig				
- Hyperplastic interstitial	-	-	+++	++
- Necrosis	-	-	+++	+
- Hyalinization	-	-	++	-
- Edema	-	-	+++	-

Score: (-) No alteration, (+) Mild alteration, (++) Moderate alteration, (+++) Severe alteration

## Discussion

Recent studies have shown that AMO influences the progress and activity of the gastrointestinal tract and changes the gut microbiota composition in the offspring of rat and pig models [19, 58]. The current study evaluated the potential role of BAC supplementation in mitigating AMO-induced gonadal toxicity in mice. Results showed that mice treated with AMO exhibited non-statistically significant variation in final body weight compared to the control group. This observation aligns with the findings stated by Dai et al. [14], who indicated that mice exposed to AMO showed no significant change in their body weight; however, it might impede physical development and diminish fatality rates. A similar conclusion was reached by Karaman et al. [30], who reported that mice treated with AMO did not exhibit any significant change in their body weight or testis weight. They explained that the minor change in the body weight and testis weight was due to the swelling of the body after the treatment with the antibiotic, and it cannot be linked to male reproductive toxicity.

Findings indicated that AMO significantly decreased male testosterone levels in plasma compared to other groups. A similar conclusion was reached by Tanyıldızı and Türk [57], who reported a decrease in the testosterone levels in male Wister rats when inducing testicular oxidative stress during exposure to imipenem, a beta-lactam antibiotic, at doses of 15, 50, and 100 mg/kg. Additionally, a similar pattern of results was obtained by Tahri et al. [55], who exhibited a decline in the plasma testosterone levels in male *Wistar* rats when exposed to cefuroxime, a beta-lactam antibiotic, at 30, 60, and 120 mg/kg. In line with previous studies, beta-lactam significantly decreased serum testosterone levels in correlation with the dose [10]. The decline in testosterone levels can be mostly attributed to the dysfunction of Leydig cells, which generate the majority of testicular testosterone [63].

Our findings are corroborated by the histopathological analysis of the testes performed in this study. We detected a decrease in spermatozoa, a reduction in spermatogenesis, apoptosis of Leydig cells, and atrophy of the seminiferous tubules following AMO treatment. These lesions are parallel with the previous findings of Fahmy et al. [18]. This adversely affects animal fertility as testosterone adjusts spermatogenesis, the activities of accessory sex organs, sexual behavior, and epididymal sperm maturation [51, 53]. Even though beta-lactam exposure caused a significant decline in the testis weight of male Wistar rats in other studies [10, 55]. Results showed a non-significant decrease in the GSI values. This observation could be attributed to the deposition of inflammatory exudates in the testes, despite the significant histopathological changes and damage observed in rats treated with 100 mg/kg, as reported by Cherif et al. [10].

Oxidative stress, the imbalance between antioxidants and free radicals, causes considerable damage to male reproductive organs. This damage originates from the oxidation of cell membrane lipids, DNA, and proteins, resulting in adverse alterations in sperm parameters and infertility [60]. It was indicated that AMO significantly decreased SOD and GSH, and increased MDA and CAT levels compared to other groups. These results were consistent with the findings of Cherif et al. [10], who exhibited a substantial increase in the testicular MDA and CAT levels in adult male *Wistar* rats treated with beta-lactam antibiotic, indicating an adaptive response to ROS formation due to oxidative stress with an increased level of H_2_O_2_. However, they observed an increase in their SOD level, which disagreed with the present findings. This conflict could be attributed to the difference in the administered dosages. Consequently, the oxidative abnormalities identified in this study enhance the formation of free radicals in intracellular testicular components, potentially reaching lethal levels that result in cellular death and organ dysfunction, affecting gene expression analysis.

Oxidative stress has been associated with cytotoxic and genotoxic consequences, and recent evidence suggests that the interaction between the DNA damage response and the mTOR pathway is the primary approach adopted by the cell to cope with genotoxic and metabolic stress. Living organisms have developed various strategies to regulate their metabolism and respond to environmental stimuli, enabling survival and growth in diverse conditions. The mTOR signaling pathway in eukaryotes combines intracellular and extracellular signals, functioning as a fundamental regulator of cellular metabolism, proliferation, and survival, and is suppressed by DNA damage [35].

To our knowledge, this is the first study to investigate the expression profile of the mTOR gene in different treated groups with AMO and BAC, indicating the ameliorative effect of BAC against the toxicity of the AMO antibiotic. From the results, AMO treatment downregulated the mTOR expression level compared to the other groups, which can be explained by the AMO-induced oxidative stress leading to ROS production. It was explained that ROS-mediated oxidative stress induces apoptosis, autophagy, and DNA damage in male germ cells, which are key factors contributing to male infertility. The intricate relationship and molecular interaction between apoptosis and autophagy can be reflected at different levels, linking the signaling pathways of both processes. ROS can induce autophagy by inhibiting the PI3K/Akt pathway, hence inhibiting the mTOR pathway and promoting autophagy [52].

Results revealed that the downregulation of mTOR is associated with a decrease in testosterone levels in the plasma. It was explained that mTOR is involved in various processes associated with male fertility and spermatogenesis. It is essential for proper meiotic sex chromosome inactivation. The inhibition of mTOR leads to germ cell depletion, decreased epididymal sperm counts, abnormalities in testicular morphology, and disruption of meiosis [38]. Researchers have shown that mTOR regulates glucose utilization and redox equilibrium in Sertoli cells, playing a direct role in the nutritional maintenance of spermatogenesis [29]. Moreover, it is also involved in the maintenance and rebuilding of the blood-testis barrier, an important step in the seminiferous epithelium cycle [36, 37]. Furthermore, the mTOR pathway is a significant regulator of autophagy, and its suppression has been revealed to enhance autophagy in Leydig cells in the testes [56].

On the other hand, probiotics are vibrant microbial dietary supplements that positively influence host health by modulating the microbial equilibrium of the gastrointestinal tract [46]. In the present study, the probiotic bacterium BAC improved serum testosterone levels in the mice group treated with the AMO antibiotic. These findings align with those reported by Çiftci and Tuna [11], who disclosed an increase in testosterone levels in the rat group receiving *Lactobacillus acidophilus* probiotic. The findings were further validated by histological analysis, which showed that testicular tissues from the BAC-supplemented group demonstrated improvements in spermatogenic cells and spermatogenesis. Additionally, the normal architecture of the seminiferous tubules and interstitial Leydig cells was observed, correlating with elevated testosterone levels. A similar pattern of results was obtained by Ghoneim and Moselhy [22], who displayed an enhancement of antioxidant activity and a significant elevation in testosterone levels in rabbits fed with probiotics. Another investigation into the effect of probiotics revealed that male mice consuming lactic acid bacteria exhibited larger testes and elevated blood testosterone levels compared to control mice, with an enhancement in spermatogenesis and an increase in the number of Leydig cells. Moreover, indications of gonadal aging were reported to diminish [42].

Probiotics modulate the PI3K/AKT/mTOR signaling pathway, promoting the coordination of the immune response [20]. The administration of BAC to the AMO-exposed group upregulated the mTOR transcript level compared to that of the AMO-exposed group. These findings align with prior research indicating that mTOR is the principal negative regulator of autophagy [31, 39]. Our findings were explained by Cui et al. [12], who indicated that *Limosilactobacillus reuteri* inhibited elevated autophagy, reduced apoptosis, and enhanced antioxidant activities in mice by promoting the mTOR signaling pathway. A similar conclusion was reached by Jeong et al. [28], who reported that the probiotic *Lactiplantibacillus pentosus* activated Akt, mTOR, and NF-κB signaling pathways in rats. Nonetheless, it was observed that the administration of probiotics to a high-fat diet of mice markedly mitigated the detrimental consequences of hyperlipidemia by diminishing testicular tissue damage, elevating blood testosterone levels, and enhancing sperm parameters [26]. The beneficial effect of probiotics on increasing testosterone levels can be explained in conjunction with their hypocholesterolemic effect, which involves the metabolism of cholesterol in the synthesis of testosterone [8, 21].

The antioxidant activity of the BAC probiotic was demonstrated by a decrease in MDA and CAT levels, and an increase in SOD and GSH levels in the group treated with AMO and supplemented with BAC compared to the AMO-treated group. These results tie well with the findings of Ghoneim and Moselhy [22], who reported a significant antioxidant efficacy of the studied probiotics in rabbits, as evidenced by a decline in MDA production and an increase in SOD and GSH activities. Comparable results have also been reported in other studies involving different probiotic strains [9, 33, 50].

Our findings differ from those of Dai et al. [14], who reported the ameliorative effects of Bacillus coagulans on reproductive dysfunction induced by general gut microbial imbalance. In contrast, the present study specifically examined the protective role of *B. clausii* against amoxicillin-induced reproductive toxicity, a model with direct clinical relevance due to the common medical use of this antibiotic. Moreover, while Dai et al. focused on restoring microbial composition, our results highlight a mechanistic pathway involving the attenuation of oxidative stress, normalization of steroidogenic enzyme expression, and improvement in testicular histoarchitecture. These differences demonstrate that *B. clausii* confers reproductive protection through molecular and structural testicular recovery, thereby filling a significant gap in understanding the probiotic modulation of antibiotic-associated reproductive dysfunction.

## Conclusion

The present study demonstrates that amoxicillin administration can directly induce reproductive toxicity in male rats, evidenced by impaired testicular structure, altered steroidogenic enzyme expression, oxidative stress elevation, and reduced hormonal profiles. These findings indicate a causal relationship between amoxicillin exposure and testicular dysfunction under the experimental conditions applied. Supplementation with *Bacillus clausii* effectively attenuated these adverse effects, suggesting its potential as a protective probiotic strategy against antibiotic-associated oxidative and reproductive disturbances through the enhancement of antioxidant activity and activation of the mTOR pathway. To our knowledge, this is the first report demonstrating a mechanistic link between probiotic intervention and mTOR-mediated protection against antibiotic-induced testicular damage. Histopathological examination confirmed the link between beta-lactam antibiotics and reproductive toxicity. These findings provide experimental evidence supporting the potential therapeutic value of *B. clausii* in counteracting antibiotic-associated reproductive disturbances. Further studies, particularly clinical investigations, are required to determine the translational relevance of these findings to human health.

## Data Availability

No datasets were generated or analysed during the current study.
